# Post-partum depression: From clinical understanding to preclinical assessments

**DOI:** 10.3389/fpsyt.2023.1173635

**Published:** 2023-04-18

**Authors:** Lorrane K. S. Moreira, Caroline V. L. Moreira, Carlos H. X. Custódio, Matheus L. P. Dias, Daniel A. Rosa, Marcos L. Ferreira-Neto, Eduardo Colombari, Elson A. Costa, James O. Fajemiroye, Gustavo R. Pedrino

**Affiliations:** ^1^Institute of Biological Sciences, Federal University of Goiás, Goiania, GO, Brazil; ^2^Department of Physiology, Institute of Biomedical Sciences, Federal University of Uberlândia, Uberlândia, Brazil; ^3^Department of Physiology and Pathology, School of Dentistry, São Paulo State University (UNESP), Araraquara, Brazil; ^4^Graduate Program in Pharmaceutical Sciences, Campus Arthur Wesley Archibald, Evangelical University of Goiás, Anápolis, Brazil

**Keywords:** depression, pharmacological targets, preclinical assessments, behavioral models, post-partum

## Abstract

Post-partum depression (PPD) with varying clinical manifestations affecting new parents remains underdiagnosed and poorly treated. This minireview revisits the pharmacotherapy, and relevant etiological basis, capable of advancing preclinical research frameworks. Maternal tasks accompanied by numerous behavioral readouts demand modeling different paradigms that reflect the complex and heterogenous nature of PPD. Hence, effective PPD-like characterization in animals towards the discovery of pharmacological intervention demands research that deepens our understanding of the roles of hormonal and non-hormonal components and mediators of this psychiatric disorder.

## 1. Introduction

The development of psychological disorders is associated with demanding physical and psychological changes during pregnancy and the postpartum period (1). Childbirth often increases the risk of mental disorders as well as higher rates of hospitalization and psychiatric treatment (2, 3). The global impact of post-partum depression (PPD) has been estimated around 20% of women at peripartum – during pregnancy or post-partum period (4). The plethora of clinical manifestations in patients with PPD includes emotional tension, mood swing, guilt, agitation, insomnia, irritability, anxiety, depressive disorder, and confusion characterized by withdrawal, intrusiveness, hostility, and non-response to infant’s cues (5, 6). The infant may later suffer from the impairment of the cognitive performance, executive function, intelligence, and language development (7, 8). Poor maternal–infant bonding, impaired development, infanticide, child neglect, and negative neurodevelopment and behavior, as well as suicide tendencies, may result from untreated PPD (5, 9, 10). Stressful life events, history of psychiatric illness, drug abuse, low levels of social or partner support, unexpected pregnancy or complications, and genetic susceptibility are among PPD’s risk factors (11, 12).

A population-based cohort study has revealed the first-time experience of psychiatric disorder in many women with PPD (13). High rates of manic-depressive, obsessive–compulsive, post-traumatic stress, eating, bipolar and pain disorders often increase the recurrence risk of the post-partum episode (14–16). Complex clinical manifestations or comorbidity of PPD with other disorders pose challenges to the accurate assessment of its prevalence. A set of cross-cultural variables, differences in the perception of mental health and its stigma, differences in environmental and socioeconomic contexts remain compounding factors (17, 18).

Historically, mental illnesses associated with childbirth were considered a discrete disease entity, a toxic-confusional or delirious picture, puerperal disorders, post-partum psychoses, baby blues, puerperal fever, or milk fever (19–23). The PPD was first described with the expression “if the womb is too moist, the brain is filled with water, and the moisture running over the eyes, compels them to involuntarily shed tears” in the 13th century or thereabout (24). With years of conceptual descriptions marked by little consensual breakthroughs on the criterion for PPD, the fifth edition-text revision of the Diagnostic and Statistical Manual of Mental Disorders (DSM-V-TR) considers PPD as a major depressive disorder (MDD) of peripartum onset. Distinguishing between MDD and PDD is difficult, for example, about 80% of PPD cases are relapses of MDD; however, particularly in PPD there are gonadal hormonal changes that accompany delivery - dramatic elevation of hormones during pregnancy that drop dramatically after delivery (25). The symptoms of PPD are often identified within the first 4 weeks of delivery up to 12 months (26, 27). Early detection, proper diagnosis, and treatment are key to preventing symptom exacerbation (28). Low mood in the early postpartum period which is largely deemed “normal” with 50–80% of new mothers often makes early intervention against PPD elusive (29, 30).

Hence, leveraging etiological factors of PPD and emotional burden exacerbation to preclinical cues could engender early intervention and benefit at-risk mothers. In this manner, this minireview revisits available pharmacological interventions, etiological hypotheses of PPD, key mediators, parameters, and preclinical framework for effective screening of drugs.

## 2. Pharmacological approaches and implications

Diverse mediators of PPD that are involved in complex pathophysiological changes provide support for hormonal and non-hormonal pharmacological interventions. The current use of typical antidepressants to treat PPD suffers from limited evidence of safety and efficacy coupled with suboptimal outcomes (31, 32). Psychotherapy or antidepressants was recommended by the American Psychiatric Association as first-line treatment for mild-to-moderate PPD (33). The monoamine-based medication which sometimes comes with the risk of teratogenicity, neonatal toxicity, and/or long-term developmental impact is widely used as the first-line treatment (Table 1). Some of them are selective serotonin reuptake inhibitors (SSRIs), serotonin and norepinephrine reuptake inhibitors (SNRIs), and tricyclic antidepressants (TCA). The worrisome infant exposure to these conventional antidepressants throughout perinatal and breastfeeding periods (40–42) should ideally limit their administration in patients. According to Deligiannidis et al. in 2021, none of these monoaminergic antidepressants have specific indications for PPD (43).

**Table 1:** General considerations and reports on antidepressant-induced adverse effects during pregnancy and lactation.

Medication	Structure	General considerations and reports on adverse effects
*SSRI*		
Fluoxetine *(Prozac)*	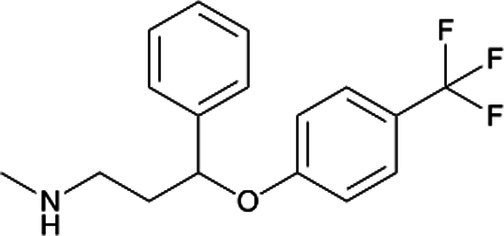	Earlier studies indicated minor malformations in neonates. Colic, seizures, irritability, withdrawal symptoms, and cyanosis during lactation. Long half-life with the stereoselective disposition of fluoxetine and norfluoxetine in the mother, fetus, breast milk, and infant
Paroxetine *(Paxil)*	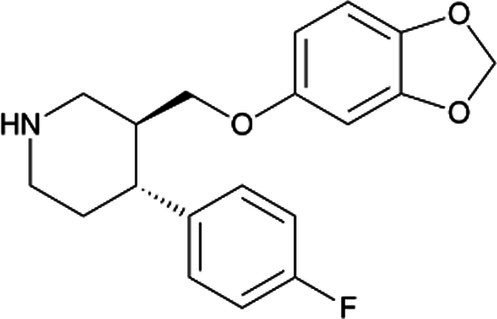	Temporary neonatal respiratory distress associated with late third trimester. Potential to increase risk in cardiovascular malformations. No adverse effects have been reported during lactation. With short half-life and high protein binding difficulty accounts for its small excretion in milk or negligible infant serum concentrations
Fluvoxamine *(Luvox)*	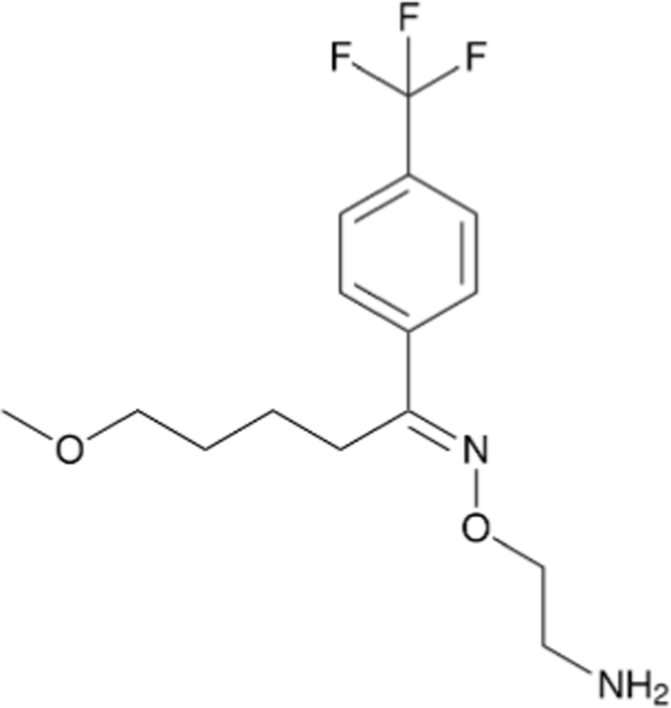	With limited data, it is not associated with an increased risk of malformations, lower birth weights, or younger gestational age and adverse effects. Shortest half-life among all SSRIs
Sertraline *(Zoloft)*	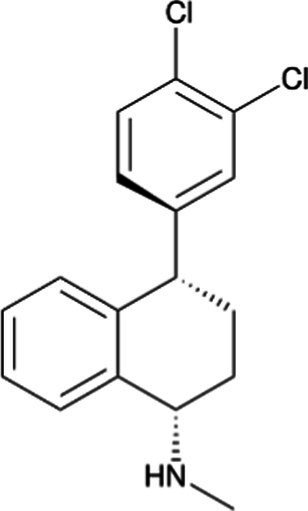	Not associated with increased risk of malformations, lower birth weights, or younger gestational age and adverse effects. Negligible effect on platelet serotonin transport in breastfed infants
Citalopram *(Celexa)*	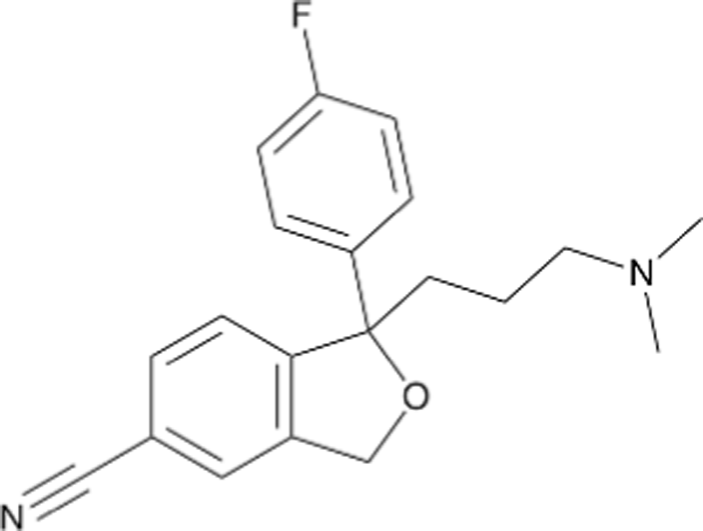	Rate of congenital anomalies was no higher than that for other SSRI exposures. Irritability, restlessness, uneasy sleep during lactation decreased feeding. Concentrations in milk is higher than in maternal plasma
Escitalopram *(Lexapro)*	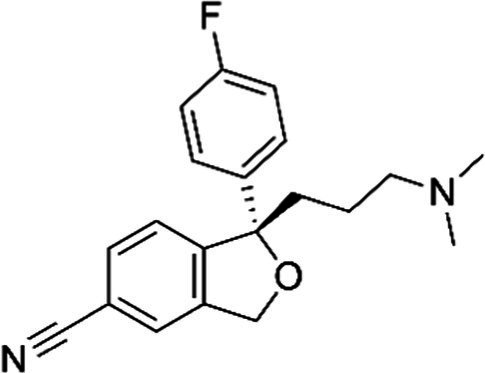	Active *S*-isomer of citalopram. Low potential for causing adverse effects as limited information indicates that maternal doses produce low levels of milk
*SNRI*		
Venlafaxine *(Effexor)*	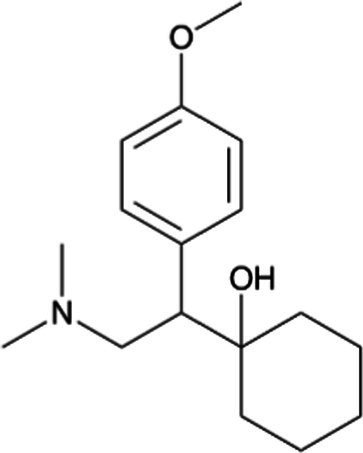	Infants receive venlafaxine and its active metabolite in breastmilk; however, concurrent side effects have rarely been reported. Breastfed infants should be monitored for excessive sedation and adequate weight gain with moderate effects of neonatal withdrawal syndrome in infants exposed to the drug during pregnancy
Desvenlafaxine *(Pristiq)*	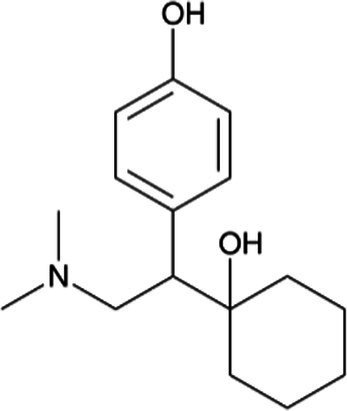	Venlafaxine’s metabolite. Neonates exposed late in the third trimester may require respiratory support. Breastfed infants should be monitored for excessive sedation and adequate weight gain
Milnacipran *(Savella)*	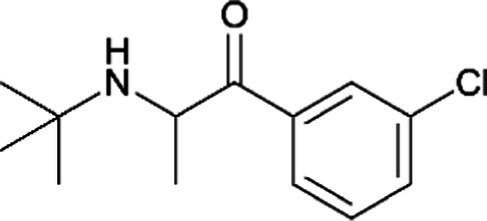	Unavailable data
*Tricyclic antidepressants*		
Nortriptyline *(Aventyl)*	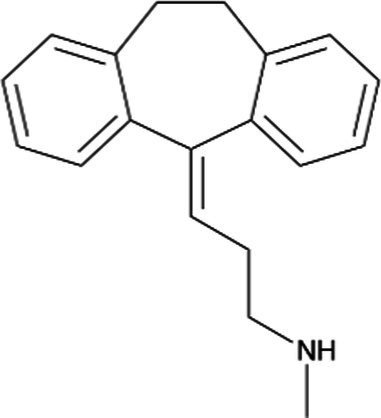	Less anticholinergic, consequently less orthostatic hypotension and constipation
Imipramine *(Trofanil)*	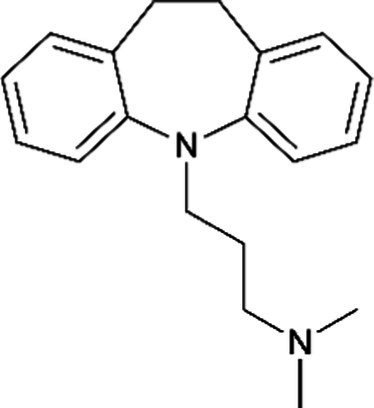	There have been clinical reports of congenital malformations. Limited data suggest that imipramine is likely to be excreted in human breast milk
*Monoamine oxidase inhibitors*		
Isocarboxazid *(Marplan)*	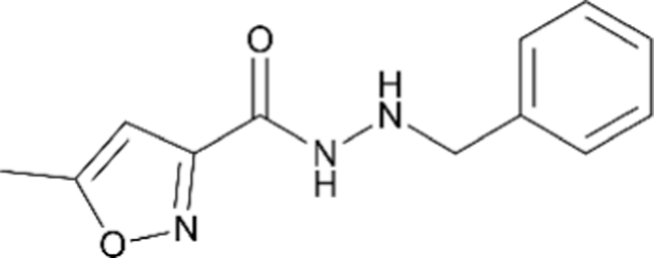	Interacts with some medications and foods to cause a life-threatening hypertensive crisis
Phenelzine *(Nardil)*	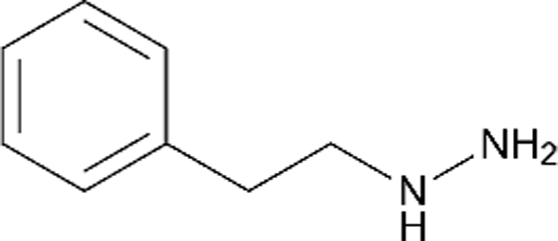	Interacts with some medications and foods to cause a life-threatening hypertensive crisis
*Others*		
Trazodone *(Donaren)*	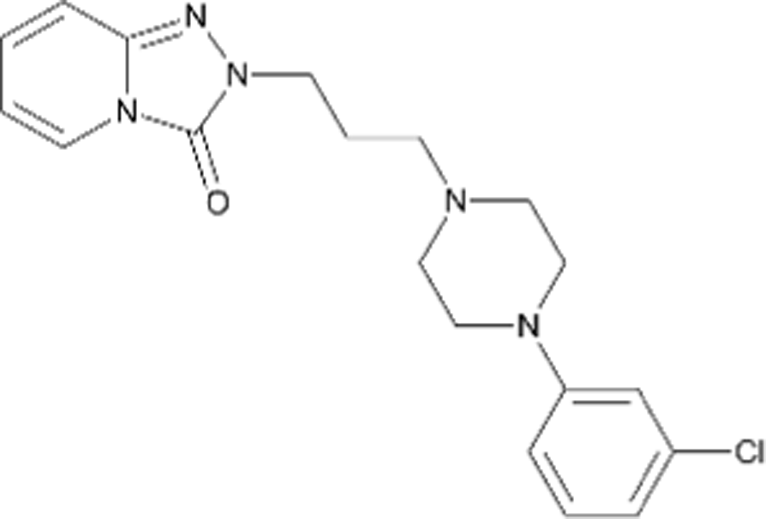	No differences in the rate of major malformations
Nefazodone *(Serzone)*	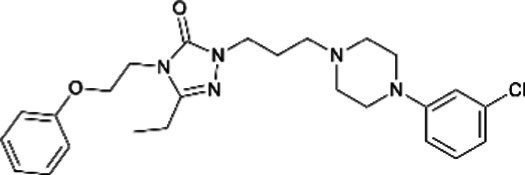	No differences in the rate of major malformations
Mirtazapine *(Remeron)*	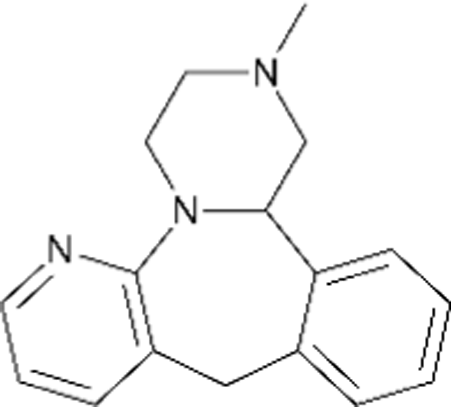	No adverse effects in infants have been reported
Bupoprion *(Wellbutrin)*	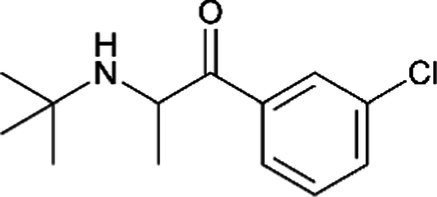	Increased risk of seizure, especially in those with a history of seizure. Limited information indicates that maternal bupropion daily produces low levels in breastmilk. Hence, more information about its use is needed

Some of these reports still have limited clinical trials, or child outcomes, and underpowered samples. SSRIs, selective serotonin reuptake inhibitors; SNRIs, serotonin norepinephrine reuptake inhibitors (34–39). All structures were designed from the name found in literature data using the ChemDraw JS program.

The etiological implication of the GABAergic system (44) through allopregnanolone [3α, 5α-tetrahydroprogesterone; a modulator of *γ*-aminobutyric acid (GABA) A receptor] provides additional insights into the drug development program for PPD. Negative feedback involving this neuroactive steroid rapidly suppresses neuronal excitability and HPA axis responses to postnatal stress to restore homeostasis (45, 46). Besides allopregnanolone, 3α, 5β-tetrahydro progesterone (pregnanolone), 5α, 3α-tetrahydrodeoxycorticosterone, pregnenolone, progesterone, and deoxycorticosterone are important neuroactives of pharmacological interest. The hypothesis supporting neurosteroids and GABAergic transmission in PPD favors clinical applications of synthetic allopregnanolone (brexanolone) and the development of zuranolone now in the third phase of the clinical trial (1). Brexanolone induces a positive allosteric modulation of GABA receptors. The activation of these receptors prior to chloride ion influx reduced the depression score. In addition, possible impairment of episodic memory has been reported in some women receiving intravenous brexanolone (47). Reducing GABA signaling has been suggested to precede exacerbated glutamatergic transmission that contributes to depressive phenotype. The approval of ketamine, a non-competitive NMDA receptor antagonist, for treatment-resistant depressive patients can support the idea of a promising effect on PPD (48, 49). Furthermore, the neurosteroid-induced GABA A receptor (GABA_A_R) modulation of the hypothalamic–pituitary-gonadal axis (50) provides a clue on the reduction in the plasma levels of luteinizing and follicle-stimulating hormone without estradiol or progesterone alteration. Hence, direct activation of GABA receptors plays important role in hormonal oscillations and regulations.

Cortisol, corticotropin-releasing hormone (CRH), adrenocorticotropic hormone (ACTH), oxytocin, prolactin, testosterone, and/or estradiol provide important pharmacological clues. Transdermal estrogen and estradiol are considered the effective treatment of depressive episodes in women with PPD or perimenopause. As the outcome of estradiol treatment raised expectations (51, 52), a transdermal estrogen for severe PPD treatment elicited more rapid improvement in depressive symptoms using the Edinburg Postnatal Depression Scale (53). However, the effectiveness and acceptability of these two treatment protocols vary among patients. The administration and abrupt withdrawal of a high dose of estradiol and progesterone during ovarian suppression in euthymic non-pregnant women increased depressive symptoms in women with a history of PPD (54). The leuprolide-induced hypogonadal post-partum hormonal changes simulation was reversed among 62% of the women with a history of PPD by supraphysiological doses of estradiol and progesterone (55). Altogether, these results reiterate how relevant hormonal targets are key to the understanding of meaningful pharmacological intervention in drug screening and development programs.

## 3. Etiological understanding and relevant mediators

**Figure 1:**
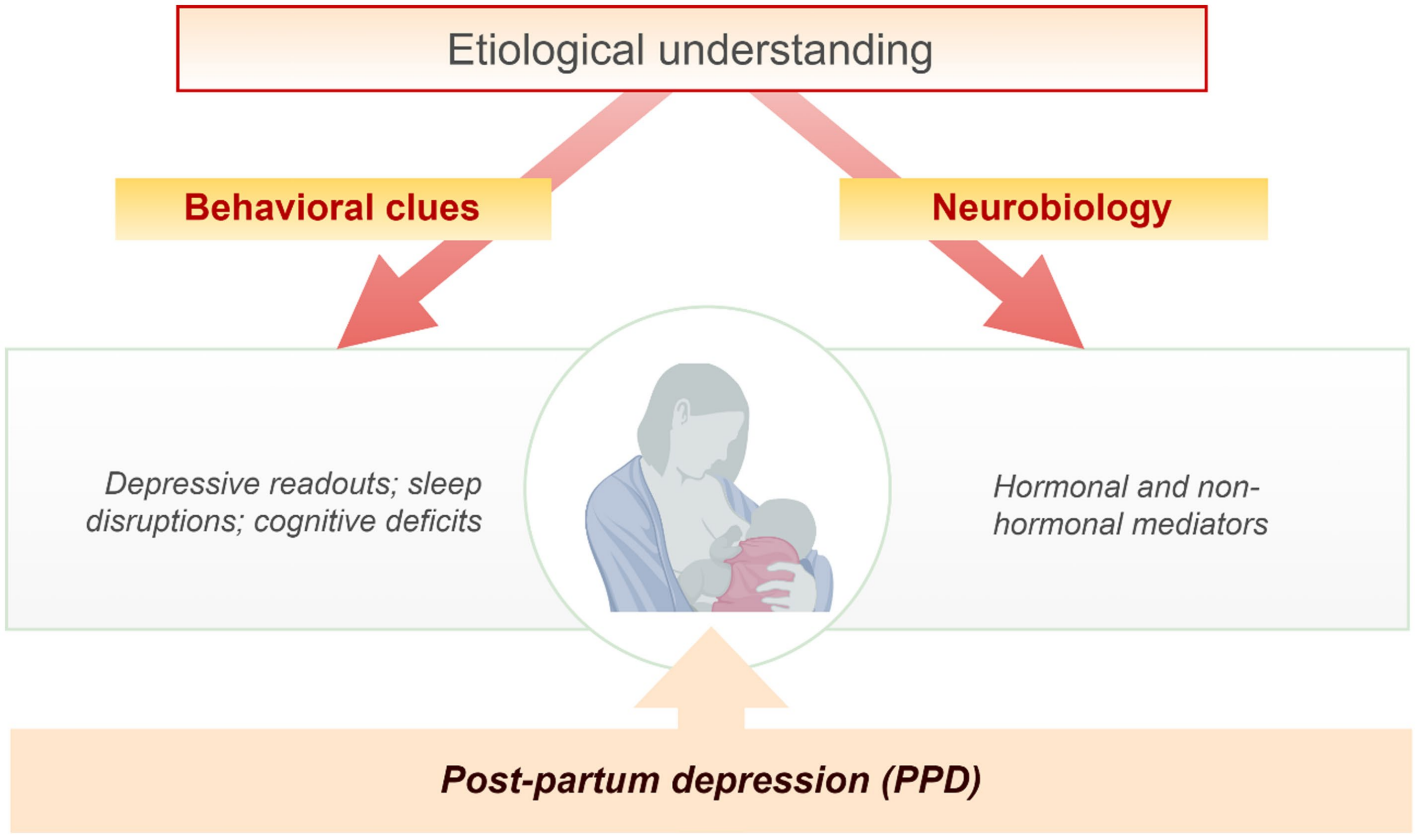
Etiological understanding of post-partum depression (PPD). The behavioral clues and neurobiological aspects suggestive of depressive signs, sleep, and cognitive disruptions, hormonal and non-hormonal alterations are important components of PPD etiology.

**Figure 2:**
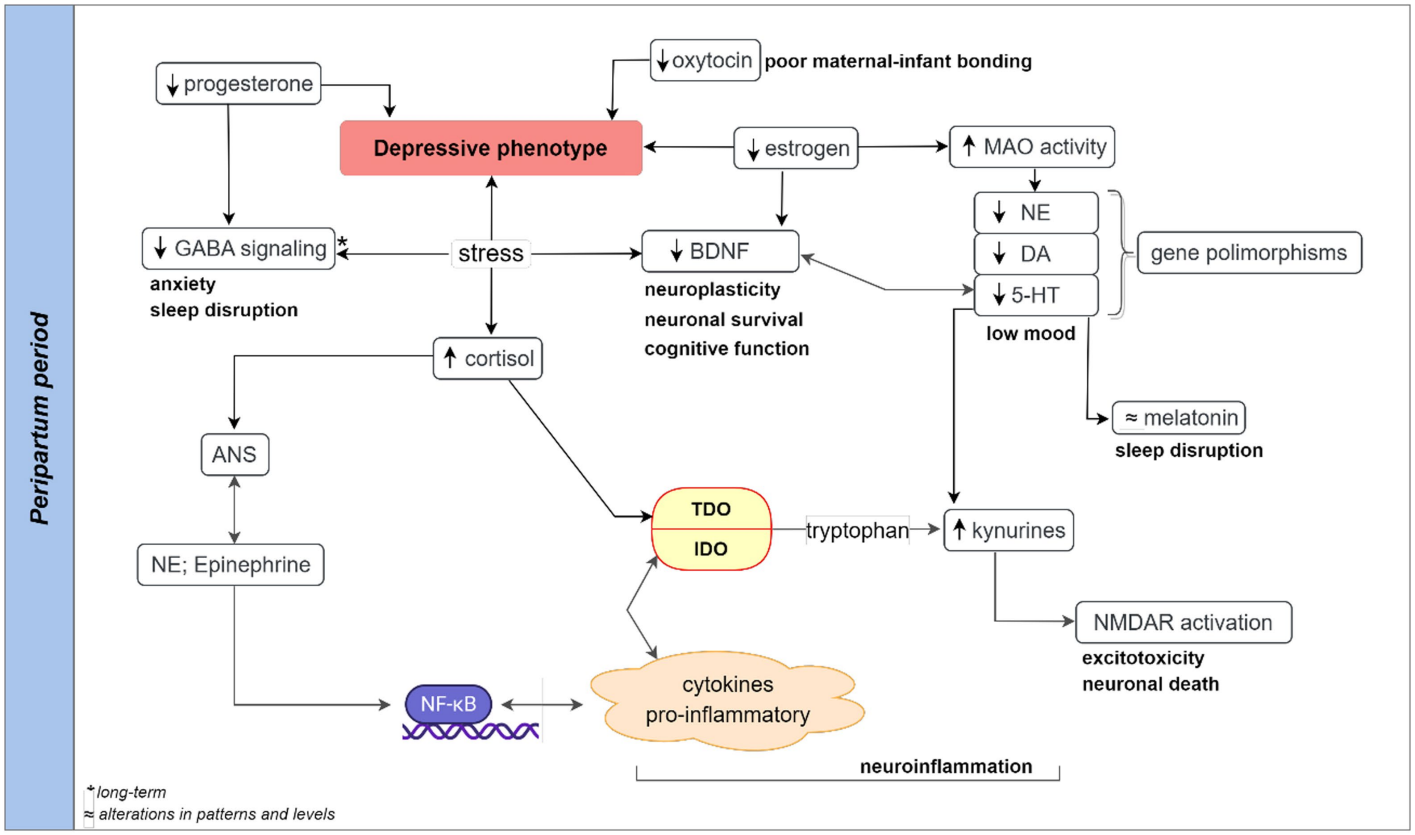
Hypothetical pathways contributing to peripartum depressive readouts. Stress-induced activation of the HPA axis could lead to a chain of events involving cortisol release and autonomic nervous system (ANS) release of catecholamines. Both HPA and ANS are highly coordinated and interconnected with the overall impact on neuroendocrine (reduced levels of progesterone, oxytocin, and estrogen), monoamines (norepinephrine, serotonin, and dopamine), and neuroinflammation (increased level of inflammatory cytokines) at the peripartum period. Long-term stress and reduced progesterone may lead to decreased GABAergic signaling, contributing to peripartum sleep disruption and anxiety. A high level of stress-induced immune-inflammatory activity could shift tryptophan-serotonin-melatonin metabolism to metabolites-driven kynurenine pathway. The downregulation of serotonin and melatonin synthesis coupled with the upregulation of kynurenic and quinolinic acid contribute to the depressive phenotype. The alterations in the tryptophan-kynurenine pathway can lead to excessive activation of N-Methyl-D-Aspartate receptor, inflammation, oxidative stress responses, and excitotoxicity leading to cellular damage, and depressive readouts in postpartum depression. A monoamine-lowering process with genetic polymorphism or a dramatic elevation of MAO-A following estrogen decline could potentiate stress-induced BDNF reductions increase the risk of neuronal damage and low mood. 5-HT, serotonin; ANS, autonomous nervous system; BDNF, brain-derived neurotrophic factor; DA, dopamine; GABA, gamma aminobutyric acid; HPA, hypothalamic–pituitary–adrenal; IDO, indoleamine 2,3-dioxygenase enzyme; MAO, Monoamine oxidase enzyme; NE, norepinephrine; NF-κB, nuclear factor kappa B; NMDAR, N-Methyl-D-Aspartate receptor; TDO, tryptophan 2,3-dioxygenase enzyme.

The behavioral cues and neurobiological aspects suggestive of depressive signs, sleep, and cognitive disruptions, and hormonal and non-hormonal alterations are important components of PPD (Figure 1). Some of these etiological components of PPD and infant suffering are the outcome of maternal exposure to stress. For instance, stress-induced reduction in circulating allopregnanolone at birth could in turn reduce its production in the neonate’s brain with far-reaching childhood adversity and multigenerational consequences (56). The stressful and intense demands of caring for a newborn are often accompanied by psychological, social, and biological factors (26, 57). Qualitative and quantitative analysis of hormonal and non-hormonal input to PPD mediated by stress is pertinent (Figure 2) as stress adaptation and resilience instinct affect the perception of uncontrollable/unpredictable events from the ones that are or become controllable/predictable. Hypothetically, stress-induced suppression of the inherent rewarding component of the maternal-newborn buffering bond could drive depression and behavioral deficits because of the disruptions of the maternal resilience and recovery system. The psychosocial hypothesis supports PPD being an endpoint of excessive worry and abrupt perinatal changes in the immunity and physiological state emanating from adapting to the maternal role and life with a new infant (57, 58). Psychosocial predictors impacting women’s resilience and mental health recovery seem to be associated with genetic components as supported by family and twin studies (59–61). These studies among others identified relevant genes that are associated with depressive episodes (60–62).

Genetic variants in hormones, transporters, receptors, and metabolic enzymes contributing to behavioral changes have been detected and analyzed during the post-partum period. Genetic alterations in estradiol, oxytocin, glucocorticoid, and CRH, estrogen receptors, and maternal gene expression across multiple brain regions with altered immune transcriptomic landscape and activation were associated with PPD (63–68). The variants of the serotonin transporter (SLC6A4; SERT), dopa decarboxylase, and protein kinase C beta play roles in stressful life events, depression, and PPD onset (69–72). High expression and polymorphism of SERT that are more reactive to environmental stressors (5-HTTLPR and STIN2-VNTR), 5-HT_2A_ receptor, tryptophan hydroxylase (TPH), neurotrophic factors, Val158Met COMT or MAO-A, as well as tryptophan depletion, could contribute to a net decrease in brain 5-HT bioavailability prior to depressive phenotype (69–73).

Physiological adaptations required for gestation undoubtedly involve neuroendocrine fluctuations at different perinatal phases (74) and may converge to set the mother’s mental health state (75, 76). This may be part of a bidirectional feedback loop settled on some hormonal axes during the peripartum period that results in persistent anxiety and depression in some women (77, 78). Over the years depressive episodes have increased research hypotheses on monoaminergic neurotransmission. Monoamines seem to be affected by hormonal changes, as revealed by the substantial modification in monoamine oxidase kinetics through a short postpartum timeframe. Robust and accelerated degradation of neurotransmitters in PPD (79) supports monoamine involvement. Estrogen has a neuroprotective role, reduces inflammatory responses, also, can improve serotonin function (25). The monoamine-lowering process with a dramatic elevation of MAO-A and simultaneous estrogen decline during the first week of postpartum (79, 80), as well as tryptophan depletion or tyrosine hydroxylase inhibition (81) are all connected to low mood and stress-induced immune-inflammatory (proinflammatory cytokines) components of peripartum changes leading to PPD (Figure 2). For instance, a report showed varying peripartum tryptophan with lower plasma concentration in the second and third trimesters as compared to the first trimester, and gradual postpartum restoration after 6 weeks (82).

Additionally, sleep disruptions and cognitive decline involving complex endocrinal and psychological interplays are important etiological manifestations in PPD patients (83–89). Cognitive dysfunction may be an early symptom of a depressive episode (89, 90). The sleep initiation, maintenance, and circadian rhythm prior to depressive manifestations and/or cognitive deficits implicate alterations in melatonin levels (91, 92), estrogen, and progesterone (90, 93–101). Besides, a high level of stress-induced immune-inflammatory activity could shift tryptophan-serotonin-melatonin metabolism to a metabolites-driven kynurenine pathway (102, 103). This is followed by a significant reduction of serotonin, a substrate for the synthesis of melatonin precursor *N*-acetylserotonin, at mid-late pregnancy and the early postpartum period (104). The downregulation of serotonin and melatonin synthesis coupled with the upregulation of kynurenic and quinolinic acid contribute to depressive phenotype (105, 106). The alterations in the tryptophan-kynurenine pathway can lead to excessive activation of NMDARs, lipid peroxidation, autoimmune, inflammation, and oxidative stress responses underlining cellular damage, and PPD (5, 107–112).

Complex interactions of kynurenine, gut microbiota, and inflammatory and hormonal mediators (113) provide additional important targets in PPD. Although both indoleamine 2,3-dioxygenase (IDO) and tryptophan 2,3-dioxygenase (TDO) convert tryptophan into kynurenine, the IDO-induced tryptophan conversion contributes to maternal-fetal immune tolerance in placental villi (protecting the fetus from maternal T-lymphocyte and natural killer cell attack during pregnancy) (114) unlike TDO that is primarily induced by the stress hormone. The IDO is induced by gamma-interferon (INF-γ), and other pro-inflammatory cytokines (5). The involvement of gut microbiota in tryptophan and serotonin metabolism provides additional insight into the role of kynurenic acid in PPD. The release of pro-inflammatory cytokines could be exacerbated by the vicious cycle of the stress-induced release of catecholamine (norepinephrine and epinephrine) and activation of the nuclear factor-kappa-beta (NF-kB) cascade (Figure 2).

The modification of autonomic and neuroendocrine reactivity (115) in response to infant cries and odors may drive maternal hyper-reaction (116). While a study associated increases in stress hormone levels with the modification of a mother’s mental health (117), an alteration in the hypothalamic–pituitary–adrenal (HPA) axis around the third month of pregnancy could boost positive feedback involving placental CRH (118). The abrupt withdrawal of placental CRH a couple of days from delivery towards the re-establishment of the maternal HPA axis could contribute to the PPD phenotype (119). Therefore, peripartum HPA screening may pave the way for new PPD pharmacotherapy interventions.

In addition, a 3-4-fold increase in basal serum oxytocin (implicated in well-being and social interaction) during pregnancy (120) prior to pulsatile increase during labor (121) seems to support the pathophysiological role of the central oxytocinergic target. The sudden reductions in oxytocin levels following the delivery in PPD (122) constitute an important hypothetical mediator. The report showed that higher oxytocin levels induced by serotonin-targeted antidepressant drugs improved cognitive performance (123) and parenting (124). This finding supports cross-communication between oxytocinergic, and serotoninergic pathways often impact other systems. The synergistic effects of the serotonin system and brain-derived neurotrophic factor (BDNF) modulate neuronal development and plasticity (125). Of note, *N*-acetylserotonin mimics BDNF activating TrkB (126). Hence, a decrease in serotonin may lower TrkB activation. Stress-induced BDNF reductions might cause neuronal damage, which would in turn heighten biological vulnerability to PPD behavioral phenotypes (127).

According to the neurotrophic hypothesis, the altered activity of neurotrophins (NTs) or their receptors plays a well-defined role in depression (128–130). The nerve growth factor (NGF) and BDNF which regulates neuronal growth and survival (128, 131) play important roles during pregnancy in the process of placental angiogenesis and maturation (132, 133). After delivery, NTs continue to perform functions in both the baby and the mother. Reduction in the level of these growth factors seems to contribute to PPD. Although lower serum BDNF levels after delivery were reported in women with PPD as compared to others without PPD (134, 135), an author suggested that a marked decrease in BDNF serum levels before and after childbirth alone did not sufficiently predict PPD (104).

Throughout the perinatal period, considerable declines in BDNF occur from the first to the third trimester prior to post-partum increase (136). Decreasing levels throughout pregnancy may, in part, reflect the use of maternal BDNF by the placenta and fetus (137). An inverse relationship between BDNF and depressive symptoms as observed in the third trimester suggests that late pregnancy is the period of greatest vulnerability to BDNF-induced depression (136). Altogether, some of these reports perhaps reflect the etiological complexity of PPD.

## 4. Update on preclinical assessments of PPD *in vivo*

As pharmacological treatment of PPD is still shrouded by limited therapeutic evidence of available antidepressants, rodent models of are fundamental to drug research and effective understanding of the neurobiology of this disorder. The assessment of PPD in animals constitutes a great challenge considering the concept of the validity of pharmacological intervention producing a similar effect in both humans and animals (predictive), the similarity of the behavioral readout or phenotype of animals to the disease phenotype being assessed in human (face validity) as well as the correlation between the underlying genetic or cellular mechanisms that result in psychological dysfunction in the animal model and in the human population (construct validity) as earlier reported (138).

In laboratory animals, pregnancy, childbirth, and lactation often induce profound physiological, neuroendocrine, and behavioral changes. The neurobiological advancement and pharmacological screening of new drugs demand improved profiling of PPD-like readouts with appropriate investigational tools in animal settings (40). A manifold of temporal peripartum cellular and molecular machinery underlining tension, mood swings, irritability, anxiety, and depression make preclinical profiling of behavioral manifestations to be herculean. Comparative assessments of behavior before and after pregnancy particularly maternal interactions with the newborn are important. The highly validated forced swimming, tail suspension, open field (139), elevated plus maze, light–dark box, and sucrose preference tests are among the important set-up for behavioral profiling.

The development of depressive-like responses and its correlation with the reductions in the level of norepinephrine, dopamine, serotonin, and intermediates in the striatum (care-giving), hippocampus (cognitive function), and hypothalamus (maternal care & eating behavior) in female BALB/c during the pregnancy and post-partum periods was used to establish PPD-like behavior (139). As PPD may be associated with alterations in the level of neurotransmitters, preclinical screening of drug targeting normalization of these alterations may be a promising candidate for the treatment of PPD. In previous studies, the withdrawal of estradiol and progesterone resulted in an increase in immobility and a decrease in forced swimming (140, 141). According to Frye and Walf, intra-amygdala administration of progesterone increased the entries in the central area of the open field, increased the time spent in the open arms of the elevated plus-maze, and decreased the freezing time after foot shock (antiaversive or antianxiety-like effect) (142). All these experimental manipulations could alter neuronal ensembles and impact maternal tasks.

The hormonal oscillations (progesterone, estradiol, prolactin, oxytocin among others) that reorganize the brain make drug injections into different brain structures an interesting preclinical procedure for behavioral profiling of despair, anhedonia, anxiety, and negative affect. Chemical injections and experimental lesions of different brain regions (medial preoptic area, ventral tegmental area, *nucleus accumbens*, and arcuate nucleus) in rodents could abolish the onset of maternal behaviors, motivation network, nursing behaviors, lactation (143, 144). These experimental interventions that disrupt endocrine and neurotransmitters communication such as monoamines are important preclinical strategies. Monoamine dysregulation especially in mesocorticolimbic structures contributes to parenting deficits and maternal care (145). Parenting tasks as measured with lactations, dams’ latency to approach the pups, total dam-pup time spent in contact measured as indices of maternal behavior, licking/grooming, active nursing, nest building, self-grooming, pups out of the nest, climbing/digging, pup retrieval, handling, crouching over pups could provide complementary cues to depressive-like behavior. Disruption of maternal tasks could provide insight into postpartum-related emotional disturbances. Lactating animals and humans have a diminished physiologic reactivity to stressors (146), increased calmness, and nurturing behavior that maintains milk quality and quantity (147). Different behavioral repertoires directed toward pups are exhibited by lactating animals with suppressed HPA responses (148). After lactation stress responses or HPA reactivity are downregulated as cortisol, glucose, ACTH, and prolactin levels return to normal (148, 149). Grooming and rearing behaviors during lactation which are indicative of increased HPA activity could be measured indirectly through stress-induced anxiety and depressive phenotypes.

As estrogen directly stimulates CRH production prior to ACTH secretion (150), its low levels during lactation may therefore suppress the hypothalamic release of CRH (146). Hence, phasic hormonal (prolactin, oxytocin, estrogen, and progesterone) levels during pregnancy, at birth, and after lactation (150) could provide important cues to behavioral changes. A high level of oxytocin being maintained throughout lactation and emotional stress may be an important parameter for measuring the antistress nature of lactation. The oxytocin-induced amnesia, anxiolysis, sedation, antinociception, lower blood pressure, decreased corticosteroid levels, and increased vagal activity (34, 146) imply systemic effects with the involvement of a wide range of mediators. An additional intervention like immobilization (stressor) of these lactating animals that elevated plasma catecholamines (epinephrine and norepinephrine) (35, 146) could be explored in preclinical settings and that could explain the role of the autonomic nervous system in PPD as shown in the hypothetic Figure 2.

The analysis of maternal behavior, impulsive responses, and consummatory behavior could correlate with hormonal oscillations. The estrogen and oxytocin modulation have been associated with maternal licking, grooming, pup gathering, and nest building in rodents (36). As estrogen levels decrease, impulsive behavior increases (37). An increase in food intake and a decrease in locomotor activity in certain female rodents following ovariectomy and the subsequent depletion of estradiol and progesterone leading to weight gain support hormonal regulation of eating behavior (38). Cyclic elevations in estradiol could modulate dopamine levels in the prefrontal cortex prior to changes in impulsive behavior. Progesterone suppression of depression-like behaviors, motor, and cognitive deficits have been associated with neuroprotection that is independent of changes in general motor coordination, pain threshold, or plasma corticosterone levels (39, 151, 152).

Altogether, drug stimulation or blockade of different brain regions as well as knockout models, lesion, or tissue removal could provide important neurobiological clues towards the screening of drug candidates. The knockout mice model with *Gabrd* gene (encoding GABA_A_R δ subunit) silencing was explored to establish abnormal peripartum GABA_A_R-induced neuroplasticity and tonic inhibition (40, 153). These mice exhibited depressive-and anxiety-like phenotypes in the postpartum period complementing the increased depressive symptoms following post-partum inhibition of progesterone metabolism (44). The perinatal cognitive impairment (90), and reduced memory processing speed deficits during late pregnancy and after childbirth could be detected using step down passive avoidance test. Ovariectomized rodents instrumented with a single bipolar stimulating electrode directed into the lateral hypothalamus could be used to study anhedonia among other behavioral manifestations. Additionally, the brain-gut-microbiome axis or pre-pregnancy exposure to stress (154), whole-cell patch-clamp recording change in synaptic receptor function, pools, and plasticity could provide meaningful mechanistic data. Considering the temporal nature of peripartum changes, neuroimaging, and real-time monitoring represent promising technology for examining patterns of hormonal and neurochemical changes that discriminate PPD from physiological changes or other depressive-like episodes.

## 5. Final considerations

Despite the current knowledge of PPD, neurobiological understanding that can augment new drug screening and treatment is still at infancy. The etiological perspectives which reveal a wide range of PPD mediators partly explain the pharmacological approaches showing significant variation in the treatment response and remission in women with PPD. Reports provide consistent evidence of complex temporal changes in gene–environment, hormonal, non-hormonal, and stress-driven mediators. PPD is etiologically different from other depressive episodes which supports the necessity of specific treatment (155). The efficacy of the conventional antidepressant drugs may be largely enhanced with appropriate adjuvant treatments or evidence-based psychotherapies. Despite the recent support for GABAergic target therapy, the reports on brexanolone or progesterone’s effects in PPD patients are still few and somehow inconclusive (156). Although the dosing regimen of brexanolone takes into consideration the serum allopregnanolone concentrations at the end of pregnancy (157, 158), the long-term impact on endogenous hormonal release or effects remains largely unclear.

The dearth of extensive analysis of peripartum phasic hormonal and non-hormonal changes leading to depressive readouts in PPD still limits the scope of pharmacological interventions. The determination of the early mediators of PPD and validated screening tools for PPD are key to early intervention, and screening of new drugs. The panacea to the myriads of controversy that are associated with the study of psychiatric disorders in animals (159, 160) includes analyses of the underlying factors of behavioral changes using a new and well-validated animal model.

An increase in immobility time that indicate depressive-like readout in rodents in the most widely used forced swimming and tail suspension tests (161, 162) could turn out to be an adaptive coping response to stress rather than behavioral despair (163). In the case of drug treatment (amphetamine), an increase in the swimming time could be an indication of CNS stimulation rather than antidepressant effects. Hence, complementary assessments of different dimensions of the depressive state of PPD as characterized by complex, heterogeneous, and multiple factors (154) could eliminate false positive or erroneous interpretations of findings. Robust assessment of the key neurobiological pathways and mediators of PPD could help in the discovery of standard pharmacological intervention.

## Author contributions

JF, EC, and GP: conceptualization, design, and methodology. LM, CM, CC, MD, DR, MF-N, and EC: writing – original draft preparation, reviewing and editing. LM and JF: software, preparation, and editing of figures. All authors contributed to the article and approved the submitted version.

## Conflict of interest

The authors declare that the research was conducted in the absence of any commercial or financial relationships that could be construed as a potential conflict of interest.

## Publisher’s note

All claims expressed in this article are solely those of the authors and do not necessarily represent those of their affiliated organizations, or those of the publisher, the editors and the reviewers. Any product that may be evaluated in this article, or claim that may be made by its manufacturer, is not guaranteed or endorsed by the publisher.
